# When parasocial relationships meet food technology neophobia: understanding consumer responses to virtual influencer marketing of upcycled foods

**DOI:** 10.3389/fnut.2026.1933051

**Published:** 2026-09-10

**Authors:** Youyou Li, Xiaoya Chen

**Affiliations:** 1 Institute of International Economics and Trade, Liaoning University of International Business and Economics, Dalian, Liaoning, China; 2 Department of Business Administration, Dongshin University, Naju-si, Jeollanam-do, Republic of Korea

**Keywords:** artificial intelligence, food technology neophobia, parasocial relationships, purchase intention, upcycled foods, virtual influencers

## Abstract

**Purpose:**

This study examines how virtual influencers’ social attractiveness, perceived authenticity, and self-disclosure are associated with consumers’ purchase intention toward upcycled foods. It further investigates parasocial relationships as a mediating mechanism and food technology neophobia as a boundary condition that may constrain the translation of relational closeness into purchase intention.

**Methods:**

Data were collected through a questionnaire survey, yielding 443 valid responses. Structural equation modeling was used to test the hypothesized relationships, bias-corrected bootstrapping with 5,000 resamples was employed to assess the mediating effects, and interaction-term and simple slope analyses were conducted to examine the moderating role of food technology neophobia.

**Results:**

Social attractiveness, perceived authenticity, and self-disclosure were all positively associated with parasocial relationships, which, in turn, were positively associated with purchase intention toward upcycled foods. Parasocial relationships partially mediated the associations between all three virtual influencer characteristics and purchase intention. Food technology neophobia significantly weakened the positive association between parasocial relationships and purchase intention.

**Conclusion:**

The findings indicate that consumer responses to upcycled foods recommended by virtual influencers reflect both relationship-building processes and technology-related concerns. Social attractiveness, perceived authenticity, and self-disclosure provide complementary relational cues that support parasocial relationship formation, while food technology neophobia limits the extent to which this psychological closeness translates into purchase intention. Firms marketing upcycled foods through virtual influencers should combine coherent persona management and sustained relationship building with transparent communication about ingredient origins, processing procedures, product safety, and nutritional quality.

## 1 Introduction

Food production, processing, and distribution generate substantial quantities of residual materials that remain suitable for human consumption (1). When these resources are excluded from the food system, the land, water, energy, and labor invested in their production are wasted, while their disposal creates additional environmental pressure (2). Upcycled foods offer a means of returning such materials to the food system by transforming ingredients that might otherwise be discarded or used for low-value purposes into foods or food ingredients with renewed edible, nutritional, and economic value through selection, safety treatment, and reprocessing (3). Their central purpose is to reduce food resource waste and improve resource-use efficiency throughout the food supply chain. Consumer acceptance is therefore essential if upcycling is to develop from a production-side resource recovery practice into a market-supported form of sustainable consumption (4). Nevertheless, consumers also consider ingredient origins, processing methods, food safety, and perceived naturalness when evaluating food products (5–7). The environmental benefits of upcycled foods alone may therefore be insufficient to secure market acceptance.

Advances in artificial intelligence, computer graphics, and digital content technologies have introduced new forms of food marketing in which virtual characters participate in product communication (8). Virtual influencers typically possess stable visual identities, personal narratives, value orientations, and content styles, enabling them to publish lifestyle content, interact with audiences, and recommend products over extended periods (9). Unlike advertising characters that appear only in isolated campaigns, virtual influencers can develop relatively complete personas through continuous content production, allowing consumers to become familiar with their personalities and communication styles through repeated exposure (10). Artificial intelligence may further enhance their capacity for content generation, audience interaction, and personalized expression, making them increasingly capable of functioning as persistent digital actors in marketing communication. Previous research has primarily explained virtual influencer effectiveness through anthropomorphism, expertise, credibility, interactivity, and physical attractiveness (11, 12). Less attention has been paid to how consumers develop sustained psychological connections with artificially created personas and whether these connections are associated with their responses to the upcycled foods such influencers recommend.

This study focuses on social attractiveness, perceived authenticity, and self-disclosure because these characteristics provide three distinct but complementary relational cues. Social attractiveness reflects the extent to which a virtual influencer is perceived as likable, approachable, and worthy of continued attention (13). Perceived authenticity does not imply that consumers mistake a virtual influencer for a real human being. Rather, it concerns whether the influencer’s persona, content, and expressed values appear natural, coherent, and consistent over time (14). Self-disclosure refers to the sharing of everyday experiences, preferences, opinions, and emotional expressions that allow audiences to learn more about the influencer’s perceived personality and lifestyle (15). Social attractiveness may encourage approach and continued attention, perceived authenticity may support recognition of a stable and coherent digital identity, and self-disclosure may deepen familiarity by providing personalized information. These characteristics should therefore not be treated merely as interchangeable favorable attributes. Instead, they represent different relational functions through which consumers may become willing to approach, recognize, and gradually feel familiar with a virtual influencer.

This integration is particularly relevant to upcycled food marketing. Consumers may have limited direct experience with upcycled foods and may be uncertain about the origins of residual ingredients, the extent of reprocessing involved, and the safety, nutritional quality, or naturalness of the final product (16, 17). Under such conditions, the communicator is not simply a source of product information but also a relational cue that may shape how consumers attend to and interpret a recommendation. A socially attractive virtual influencer may encourage continued exposure to the product message (18), an authentic and coherent persona may make the recommendation appear more consistent with the influencer’s established identity (19), and self-disclosure may make the recommendation feel more personally embedded in the influencer’s ongoing narrative (20). Although previous studies have examined these characteristics separately in relation to consumer responses, less is known about how they jointly support relationship formation in the context of marketing a food innovation that may simultaneously evoke sustainability interest and processing-related concern.

Parasocial relationship theory provides a useful basis for explaining how consumers respond to virtual influencer recommendations of upcycled foods. Repeated exposure to a virtual influencer’s narratives, lifestyle content, and expressed opinions may create a one-sided sense of familiarity and psychological closeness, allowing the influencer to be perceived as a recognizable relational figure rather than merely a commercial communication device (21, 22). This relationship may increase consumers’ attention to and acceptance of product recommendations, thereby linking social attractiveness, perceived authenticity, and self-disclosure with purchase intention. However, relational closeness may not fully overcome concerns about how upcycled foods are produced. Although these products do not necessarily involve highly complex technologies, the recovery and transformation of residual food materials may still be perceived as unfamiliar or nontraditional processing (23). Consumers with stronger food technology neophobia may therefore remain cautious even when they feel close to the recommending virtual influencer (17, 24). Existing research on upcycled foods has largely emphasized product knowledge, perceived value, environmental awareness, attitudes, and risk perceptions, while studies of virtual influencers have focused more on communicator characteristics and relationship formation (16, 25–27). Less is known about how parasocial relationships and technology-related resistance operate together. This study therefore examines parasocial relationships as a mediating mechanism and food technology neophobia as a boundary condition in the association between virtual influencer characteristics and purchase intention toward upcycled foods.

To address these gaps, this study develops a moderated mediation model that examines the associations of virtual influencers’ social attractiveness, perceived authenticity, and self-disclosure with parasocial relationships, as well as the association between parasocial relationships and consumers’ purchase intention toward upcycled foods recommended by virtual influencer. The moderating role of food technology neophobia is also assessed. The study contributes to theory in three respects. First, it conceptualizes social attractiveness, perceived authenticity, and self-disclosure as complementary relational cues that support approach, identity recognition, and personal familiarity rather than treating them as isolated favorable characteristics. Second, it extends parasocial relationship theory to the context of virtual influencer communication for upcycled foods, thereby connecting digital relationship formation with the market acceptance of a sustainable food innovation. Third, it identifies food technology neophobia as a boundary condition that may constrain the conversion of parasocial closeness into purchase intention. Existing source-oriented perspectives are useful for explaining how consumers evaluate communicators, while food acceptance perspectives explain why consumers may hesitate to adopt novel products. Neither perspective alone fully explains how relationship-building characteristics of a nonhuman digital persona interact with technology-related resistance in shaping responses to upcycled food recommendations. Practically, the findings may provide guidance for upcycled food companies, virtual influencer operators, and food communication organizations seeking to combine relationship-oriented marketing with transparent communication about food processing and safety.

Accordingly, this study addresses the following research questions:

*RQ1*: Are the social attractiveness, perceived authenticity, and self-disclosure of virtual influencers positively associated with parasocial relationships?

*RQ2*: Do parasocial relationships mediate the associations between the three virtual influencer characteristics and purchase intention toward upcycled foods?

*RQ3*: Does food technology neophobia moderate the association between parasocial relationships and purchase intention toward upcycled foods?

## 2 Literature review and hypothesis development

### 2.1 Virtual influencer characteristics and parasocial relationships

The stable personas and continuous content production of virtual influencers allow consumers to develop familiarity and psychological closeness through repeated exposure. Social attractiveness makes a virtual influencer appear likable, approachable, and easy to interact with, thereby increasing consumers’ willingness to follow its content and remain informed about its activities (18). Perceived authenticity is reflected in the consistency among the influencer’s persona, expressed values, and content (28). Although consumers recognize that virtual influencers are created through artificial intelligence or other digital technologies, coherent and natural behavior may still lead them to perceive these digital characters as communication agents with stable identities and distinctive personalities, while reducing perceptions of commercial manipulation or deliberate fabrication (29). Self-disclosure further enables consumers to learn about a virtual influencer through the sharing of everyday experiences, personal interests, opinions, and emotional expressions (15). As such personalized information accumulates, consumers may increasingly feel that they know the virtual influencer, thereby reducing perceived psychological distance (30). These three characteristics therefore serve different but complementary relational functions: social attractiveness promotes approach and continued attention, perceived authenticity supports recognition of a coherent digital identity, and self-disclosure deepens personal familiarity.

These relational cues may be especially important in the context of upcycled food marketing. Consumers often have limited direct experience with upcycled foods and may be uncertain about residual ingredient sources, processing methods, food safety, nutritional quality, and perceived naturalness (31). Under such conditions, a virtual influencer’s recommendation is evaluated not only as product information but also through the relationship consumers have developed with the digital persona. Social attractiveness may encourage continued exposure to food-related content (18, 32), perceived authenticity may make the recommendation appear consistent with the influencer’s established identity and values (33), and self-disclosure may embed the recommendation within a more familiar and personalized narrative (34). Although previous studies have examined these characteristics separately as favorable attributes of virtual influencers, less attention has been paid to how they jointly provide the relational foundation for parasocial relationships in the promotion of an innovative and potentially unfamiliar food category. Accordingly, the following hypotheses are proposed:

*H1a*: The social attractiveness of a virtual influencer is positively associated with consumers’ parasocial relationship with the virtual influencer.

*H1b*: The perceived authenticity of a virtual influencer is positively associated with consumers’ parasocial relationship with the virtual influencer.

*H1c*: The self-disclosure of a virtual influencer is positively associated with consumers’ parasocial relationship with the virtual influencer.

### 2.2 Parasocial relationships and purchase intention

Parasocial relationships may increase the extent to which consumers attend to information provided by virtual influencers and incorporate their recommendations into purchase decisions. Through continued exposure, consumers may develop familiarity, psychological closeness, and emotional attachment to a virtual influencer, causing it to be perceived not merely as a digital character delivering commercial messages but as a familiar figure worthy of attention (10, 35). Such relational experiences may reduce resistance to commercial recommendations and increase consumers’ receptiveness to product information. This relationship may be particularly relevant to upcycled foods, whose ingredient origins, production processes, safety, and nutritional quality are difficult for consumers to evaluate before purchase (36). The reuse of residual food materials may also generate uncertainty. Recommendations from a virtual influencer with whom consumers have developed a strong psychological connection may provide relational cues that facilitate the interpretation and evaluation of such products, while increasing confidence in the recommendation (12). Stronger parasocial relationships may therefore be associated with greater acceptance of the virtual influencer’s product advice and higher purchase intention toward the upcycled foods it recommends (37). Accordingly, the following hypothesis is proposed:

*H2*: Consumers’ parasocial relationship with a virtual influencer is positively associated with their purchase intention toward upcycled foods recommended by that virtual influencer.

### 2.3 The mediating role of parasocial relationships

The social attractiveness, perceived authenticity, and self-disclosure of virtual influencers may not translate directly into consumers’ purchase intention toward upcycled foods. Instead, these characteristics may first shape consumers’ relational experiences with virtual influencers. Social attractiveness increases consumers’ willingness to approach and continuously follow a virtual influencer (37), perceived authenticity makes the influencer’s persona appear stable, natural, and coherent (30), and self-disclosure enables consumers to gradually learn about the influencer’s daily life, interests, preferences, and values (38). These relational cues may strengthen consumers’ familiarity and psychological closeness to the virtual influencer, thereby facilitating the development of a parasocial relationship. When consumers perceive a virtual influencer as a familiar and attention-worthy digital figure, its product recommendations are more likely to acquire relational meaning rather than being regarded merely as commercial messages (39, 40). This psychological connection may be particularly relevant to upcycled foods, whose ingredient origins and processing methods may raise consumer concerns (17). A stronger parasocial relationship may increase consumers’ attention to and acceptance of the recommendation, which may subsequently be associated with greater purchase intention (41, 42). Parasocial relationships may therefore serve as a mechanism through which the relationship-building characteristics of virtual influencers are linked to consumers’ responses to the upcycled foods they recommend. Accordingly, the following hypotheses are proposed:

*H3a*: Parasocial relationships mediate the association between the social attractiveness of virtual influencers and consumers’ purchase intention toward upcycled foods.

*H3b*: Parasocial relationships mediate the association between the perceived authenticity of virtual influencers and consumers’ purchase intention toward upcycled foods.

*H3c*: Parasocial relationships mediate the association between the self-disclosure of virtual influencers and consumers’ purchase intention toward upcycled foods.

### 2.4 The moderating role of food technology neophobia

Food technology neophobia refers to consumers’ tendency to distrust, avoid, or feel concerned about foods produced through unfamiliar or novel production, processing, and transformation technologies (43, 44). It differs from general food neophobia, which primarily reflects reluctance to try unfamiliar foods, and from product-specific perceived risk, which concerns the anticipated negative consequences associated with consuming a particular product (45, 46). Food technology neophobia is directed more specifically toward the technological processes used to produce or modify food. Consumers with higher levels of food technology neophobia are more likely to question whether the claimed benefits of new food technologies are overstated and to worry that technological processing may reduce food naturalness or generate uncertain food safety and long-term health risks (47). These concerns may persist even when the product is associated with environmental or resource-efficiency benefits.

This tendency is particularly relevant to upcycled foods, which require the selection, treatment, and reprocessing of food materials that would otherwise remain underutilized (48). Although upcycled foods do not necessarily rely on highly complex technologies, consumers may perceive the recovery and transformation of residual ingredients as a nontraditional production process and therefore associate such products with greater technological uncertainty (49). Under these conditions, even a strong parasocial relationship with a virtual influencer may not fully overcome concerns about ingredient transformation, processing safety, or long-term health consequences. Consumers with high food technology neophobia may consequently be less willing to rely on relational cues when evaluating the recommendation (50), whereas those with lower food technology neophobia are generally more open to novel food production methods and more receptive to recommendations from familiar virtual influencers (43, 51, 52). Food technology neophobia is therefore expected to weaken the extent to which parasocial relationships are translated into purchase intention toward upcycled foods. Accordingly, the following hypothesis is proposed:

*H4*: Food technology neophobia negatively moderates the association between parasocial relationships and purchase intention toward upcycled foods, such that the positive association becomes weaker at higher levels of food technology neophobia.

**FIGURE 1:**
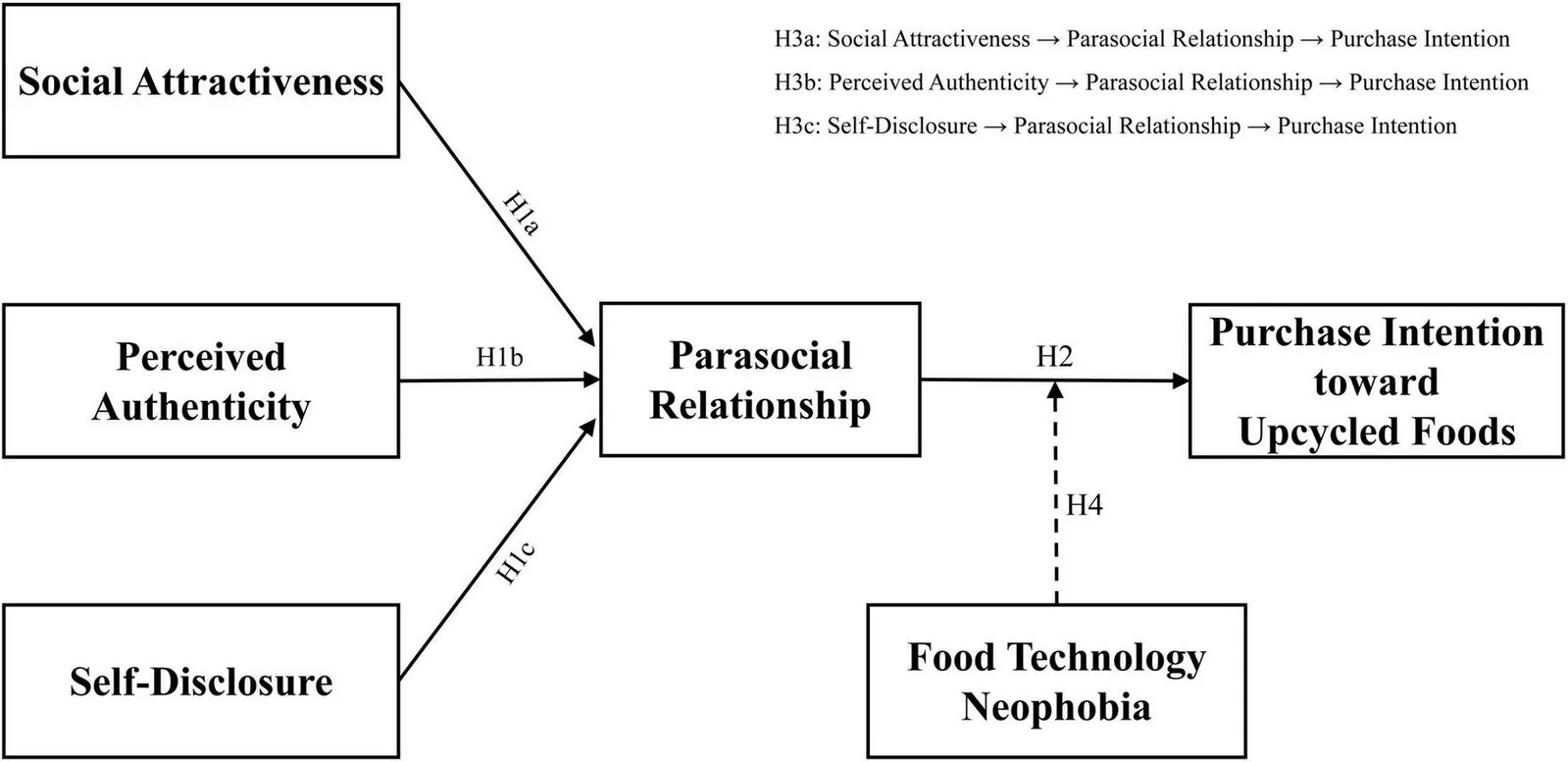
Conceptual model of virtual influencer characteristics, parasocial relationships, food technology neophobia, and purchase intention toward upcycled foods.

The proposed research model is presented in Figure 1.

## 3 Methods

### 3.1 Scale development

A structured questionnaire was developed to examine the relationships among virtual influencer characteristics, parasocial relationships, food technology neophobia, and purchase intention toward upcycled foods. The research model comprised six constructs: social attractiveness, perceived authenticity, self-disclosure, parasocial relationships, purchase intention, and food technology neophobia. To ensure measurement reliability and content validity, all items were adapted from established scales and modified to fit the context of virtual influencers recommending upcycled foods. Specifically, social attractiveness was measured using items adapted from Kim et al. (13) and Yoo et al. (53); perceived authenticity was assessed based on Vo et al. (54); self-disclosure was measured with items adapted from Kim and Kim (15); parasocial relationships were assessed using measures derived from Di Dalmazi et al. (55) and Wang et al. (56); purchase intention was measured based on Li and Shan (57) and Yin et al. (58); and food technology neophobia was assessed using items adapted from Proserpio et al. (59) and Zamparo et al. (60). The full list of measurement items is presented in Supplementary Appendix.

The questionnaire consisted of four sections. The first section introduced the purpose of the study, the voluntary nature of participation, and the principles governing data use. The second section provided brief definitions of virtual influencers and upcycled foods and included screening questions. The third section measured the focal constructs using a five-point Likert scale ranging from 1 (“strongly disagree”) to 5 (“strongly agree”). The final section collected demographic information, including gender, age, educational attainment, and monthly income. Before the formal survey, the questionnaire was translated and back-translated to ensure semantic equivalence. Researchers familiar with the relevant fields also reviewed the wording of the items to minimize potential bias arising from linguistic differences and contextual adaptations. Before the main survey, a small-scale pilot test was conducted to assess the clarity, readability, and overall structure of the questionnaire.

### 3.2 Data collection

Data were collected using a combination of judgmental and convenience sampling. The questionnaire was administered through Wenjuanxing, a Chinese online survey platform, and distributed to potential respondents through social media and consumer communities. All participants were informed of the study purpose, the anonymous nature of the survey, the voluntary nature of participation, and the intended use of the data. Eligible participants were required to be at least 18 years old, currently follow at least one virtual influencer, and have encountered content posted by that influencer within the preceding month. After passing the screening question, respondents were asked to identify the virtual influencer with whom they were most familiar or whom they followed most frequently. They were instructed to answer all subsequent questions concerning social attractiveness, perceived authenticity, self-disclosure, and parasocial relationships with reference to this focal virtual influencer. Allowing respondents to select a familiar virtual influencer provided an experience-based basis for evaluating parasocial relationships rather than requiring them to assess an unfamiliar digital character.

Before evaluating the product recommendation, all respondents received standardized explanations of virtual influencers and upcycled foods. They were then presented with a standardized text-based scenario involving a specific upcycled food product. Respondents were asked to imagine that their focal virtual influencer was recommending an upcycled apple chip product. The product was described as being made from edible apple peels and pulp remaining from apple juice production and other fruit-processing activities. These materials, which might otherwise be discarded or used for lower-value purposes, were subjected to standardized selection, drying, grinding, and food-processing procedures before being transformed into apple chips. Respondents were instructed to evaluate their purchase intention with reference to this recommendation scenario. All participants received the same information about the source of the upcycled ingredients, the main processing procedures, and the final product form. This standardized presentation was intended to reduce variation in respondents’ interpretations of the focal product and ensure that their purchase-intention judgments referred to a consistent recommendation context.

A total of 522 questionnaires were collected. Responses were excluded if they had an unusually short completion time, or exhibited clear patterns of inattentive responding. The final sample consisted of 443 valid questionnaires, corresponding to an effective response rate of 84.87%. Of the respondents, 46.50% were male and 53.50% were female. The sample covered different age groups, although respondents aged 18–35 accounted for a relatively large proportion. Educational attainment was concentrated primarily at the associate and bachelor’s degree levels, while monthly income was most commonly between RMB 3,000 and RMB 12,000. Gender, age, education, and income were collected to describe the demographic and socioeconomic composition of the sample and to provide contextual information for interpreting consumer responses to virtual influencer marketing and innovative food products. Overall, the final sample showed reasonable diversity across these demographic characteristics.

### 3.3 Data analysis

Data analysis was conducted using IBM SPSS Statistics and AMOS. The main analysis proceeded in five stages. First, the internal consistency of the measurement scales was assessed using Cronbach’s alpha and composite reliability, convergent validity was assessed using standardized factor loadings and average variance extracted, whereas discriminant validity was examined using the Fornell–Larcker criterion and the heterotrait–monotrait ratio of correlations. Second, Common method bias was evaluated using Harman’s single-factor test.

Third, structural equation modeling was used to test the hypothesized associations of social attractiveness, perceived authenticity, and self-disclosure with parasocial relationships, as well as the association between parasocial relationships and purchase intention toward upcycled foods. Fourth, the mediating role of parasocial relationships was examined using bias-corrected bootstrapping with 5,000 resamples and 95% confidence intervals. An indirect effect was considered statistically significant when its confidence interval did not include zero.

Fifth, the moderating role of food technology neophobia was assessed using an interaction-term analysis. Parasocial relationships and food technology neophobia were mean-centered before their interaction term was created to reduce nonessential multicollinearity. Simple slope analysis was then conducted to estimate the association between parasocial relationships and purchase intention at low, mean, and high levels of food technology neophobia, represented by one standard deviation below the mean, the mean, and one standard deviation above the mean, respectively.

## 4 Results

### 4.1 Reliability and validity

The reliability and convergent validity of the measurement model were assessed using factor loadings, composite reliability, average variance extracted, and Cronbach’s alpha. As shown in Table 1, the standardized factor loadings ranged from 0.713 to 0.805, all exceeding the recommended threshold of 0.70. This indicates that the observed items adequately represented their respective constructs. Composite reliability values ranged from 0.828 to 0.904 and were therefore above the recommended value of 0.70, demonstrating satisfactory internal consistency. The average variance extracted values ranged from 0.546 to 0.610, all exceeding 0.50, indicating that each latent construct explained more than half of the variance in its corresponding indicators. The measurement model therefore demonstrated satisfactory convergent validity. In addition, Cronbach’s alpha values ranged from 0.827 to 0.904, further confirming the internal reliability of the measurement scales.

**TABLE 1:** Reliability and convergent validity of the measurement model.

Construct	Code	Factor loading	Cronbach’s α	AVE	CR
Social attractiveness	SA1	0.713	0.829	0.549	0.829
	SA2	0.773			
	SA3	0.743			
	SA4	0.734			
Perceived authenticity	PA1	0.725	0.827	0.546	0.828
	PA2	0.729			
	PA3	0.769			
	PA4	0.731			
Self-disclosure	SD1	0.767	0.830	0.551	0.831
	SD2	0.737			
	SD3	0.745			
	SD4	0.718			
Parasocial relationship	PSR1	0.778	0.881	0.598	0.881
	PSR2	0.752			
	PSR3	0.762			
	PSR4	0.805			
	PSR5	0.769			
Purchase intention	PI1	0.788	0.859	0.604	0.859
	PI2	0.743			
	PI3	0.787			
	PI4	0.790			
Food technology neophobia	FTN1	0.779	0.904	0.610	0.904
	FTN2	0.796			
	FTN3	0.790			
	FTN4	0.782			
	FTN5	0.761			
	FTN6	0.777			

AVE, average variance extracted; CR, composite reliability.

Discriminant validity was further assessed using the Fornell–Larcker criterion and the heterotrait–monotrait ratio of correlations. As shown in Table 2, the square roots of the AVE values for social attractiveness, perceived authenticity, self-disclosure, parasocial relationships, purchase intention, and food technology neophobia were 0.741, 0.739, 0.742, 0.773, 0.777, and 0.781, respectively. Each value exceeded the correlations between the corresponding construct and all other constructs. As reported in Table 3, the HTMT values ranged from 0.104 to 0.525, all below the recommended threshold of 0.85. Thus, both the Fornell–Larcker criterion and the HTMT results supported the discriminant validity of the measurement model.

**TABLE 2:** Pearson correlations and discriminant validity based on the Fornell–Larcker criterion.

Construct	SA	PA	SD	PSR	PI	FTN
SA	0.741	0.739	0.742	0.773	0.777	0.781
PA	0.333					
SD	0.369	0.392				
PSR	0.434	0.447	0.419			
PI	0.395	0.366	0.324	0.386		
FTN	−0.132	−0.135	−0.089	−0.156	−0.180	

Diagonal values in bold represent the square roots of the AVE values. SA, social attractiveness; PA, perceived authenticity; SD, self-disclosure; PSR, parasocial relationship; PI, purchase intention; FTN, food technology neophobia.

**TABLE 3:** Discriminant validity assessment using the heterotrait–monotrait ratio.

Construct	SA	PA	SD	PSR	PI	FTN
SA	–	–	–	–	–	–
PA	0.401					
SD	0.446	0.472				
PSR	0.508	0.525	0.490			
PI	0.467	0.434	0.384	0.444		
FTN	0.152	0.155	0.104	0.177	0.205	

SA, social attractiveness; PA, perceived authenticity; SD, self-disclosure; PSR, parasocial relationship; PI, purchase intention; FTN, food technology neophobia.

### 4.2 Common method bias

Because the independent variables, mediator, moderator, and dependent variable were measured using the same questionnaire at a single point in time, the data may be susceptible to common method bias. Harman’s single-factor test was therefore conducted by entering all measurement items into an unrotated exploratory factor analysis. The results showed that the first factor accounted for 28.186% of the total variance, well below the commonly used threshold of 50%, and no single factor explained the majority of the variance. These results suggest that common method bias was unlikely to pose a serious threat to the subsequent statistical analyses.

### 4.3 Structural equation modeling

Structural equation modeling was used to examine the associations of social attractiveness, perceived authenticity, and self-disclosure with parasocial relationships, as well as the association between parasocial relationships and purchase intention toward upcycled foods. The structural model demonstrated a satisfactory fit to the data: χ^2^/df = 2.213, GFI = 0.918, RMSEA = 0.052, SRMR = 0.063, CFI = 0.949, NFI = 0.912, and NNFI = 0.941. All fit indices met the recommended criteria, indicating that the proposed model was adequately consistent with the observed data.

As shown in Table 4, social attractiveness was positively associated with parasocial relationships (β = 0.301, *p* < 0.001), supporting H1a. Perceived authenticity was also positively associated with parasocial relationships (β = 0.316, *p* < 0.001), supporting H1b. Self-disclosure showed a significant positive association with parasocial relationships (β = 0.217, *p* < 0.001), thereby supporting H1c. Among the three virtual influencer characteristics, perceived authenticity exhibited the strongest association with parasocial relationships, followed by social attractiveness and self-disclosure. In addition, parasocial relationships were positively associated with purchase intention toward upcycled foods (β = 0.476, *p* < 0.001). This result indicates that stronger psychological connections with virtual influencers are associated with greater intention to purchase the upcycled foods they recommend. H2 was therefore supported.

**TABLE 4:** Structural model results and hypothesis testing.

Hypothesis	Path	*z*	*p*	β
H1a	SA → PSR	5.252	0.000	0.301
H1b	PA → PSR	5.430	0.000	0.316
H1c	SD → PSR	3.723	0.000	0.217
H2	PSR → PI	8.552	0.000	0.476

SA, social attractiveness; PA, perceived authenticity; SD, self-disclosure; PSR, parasocial relationship; PI, purchase intention.

### 4.4 Mediating effects

The mediating role of parasocial relationships was examined using bias-corrected bootstrapping with 5,000 resamples. The significance of the three indirect effects was assessed using 95% bootstrap confidence intervals. An indirect effect was considered significant when the corresponding confidence interval did not include zero.

As shown in Table 5, the indirect effect of social attractiveness on purchase intention through parasocial relationships was 0.047, with a 95% bootstrap confidence interval of [0.020, 0.084]. Because the confidence interval did not include zero, the indirect effect was significant. The direct effect of social attractiveness on purchase intention also remained significant (direct effect = 0.244, *p* < 0.01), indicating partial mediation. H3a was therefore supported.

**TABLE 5:** Bootstrap results for the mediating effects of parasocial relationships.

Path	Total effect	Indirect effect	z	95% BootCI	Direct effect
SA → PSR → PI	0.291**	0.047	2.919	[0.020, 0.084]	0.244**
PA → PSR → PI	0.249**	0.051	2.972	[0.022, 0.090]	0.198**
SD → PSR → PI	0.147**	0.038	2.636	[0.016, 0.073]	0.109*

BootCI, bootstrap confidence interval; SA, social attractiveness; PA, perceived authenticity; SD, self-disclosure; PSR, parasocial relationship; PI, purchase intention. *p < 0.05, **p < 0.01.

The indirect effect of perceived authenticity on purchase intention through parasocial relationships was 0.051, with a 95% bootstrap confidence interval of [0.022, 0.090]. This confidence interval also excluded zero. Because the direct effect of perceived authenticity on purchase intention remained significant (direct effect = 0.198, *p* < 0.01), parasocial relationships partially mediated this association. H3b was supported.

The indirect effect of self-disclosure on purchase intention through parasocial relationships was 0.038, with a 95% bootstrap confidence interval of [0.016, 0.073]. The confidence interval did not include zero, indicating a significant indirect effect. The direct effect of self-disclosure on purchase intention also remained significant (direct effect = 0.109, *p* < 0.05), demonstrating partial mediation. H3c was therefore supported.

### 4.5 Moderating effect

This study examined whether food technology neophobia moderated the association between parasocial relationships and purchase intention toward upcycled foods. Parasocial relationships and food technology neophobia were mean-centered before the interaction term was created. The results showed that the interaction between parasocial relationships and food technology neophobia was negatively associated with purchase intention toward upcycled foods (*t* = −3.986, *p* < 0.001). This finding indicates that food technology neophobia weakened the positive association between parasocial relationships and purchase intention.

**FIGURE 2:**
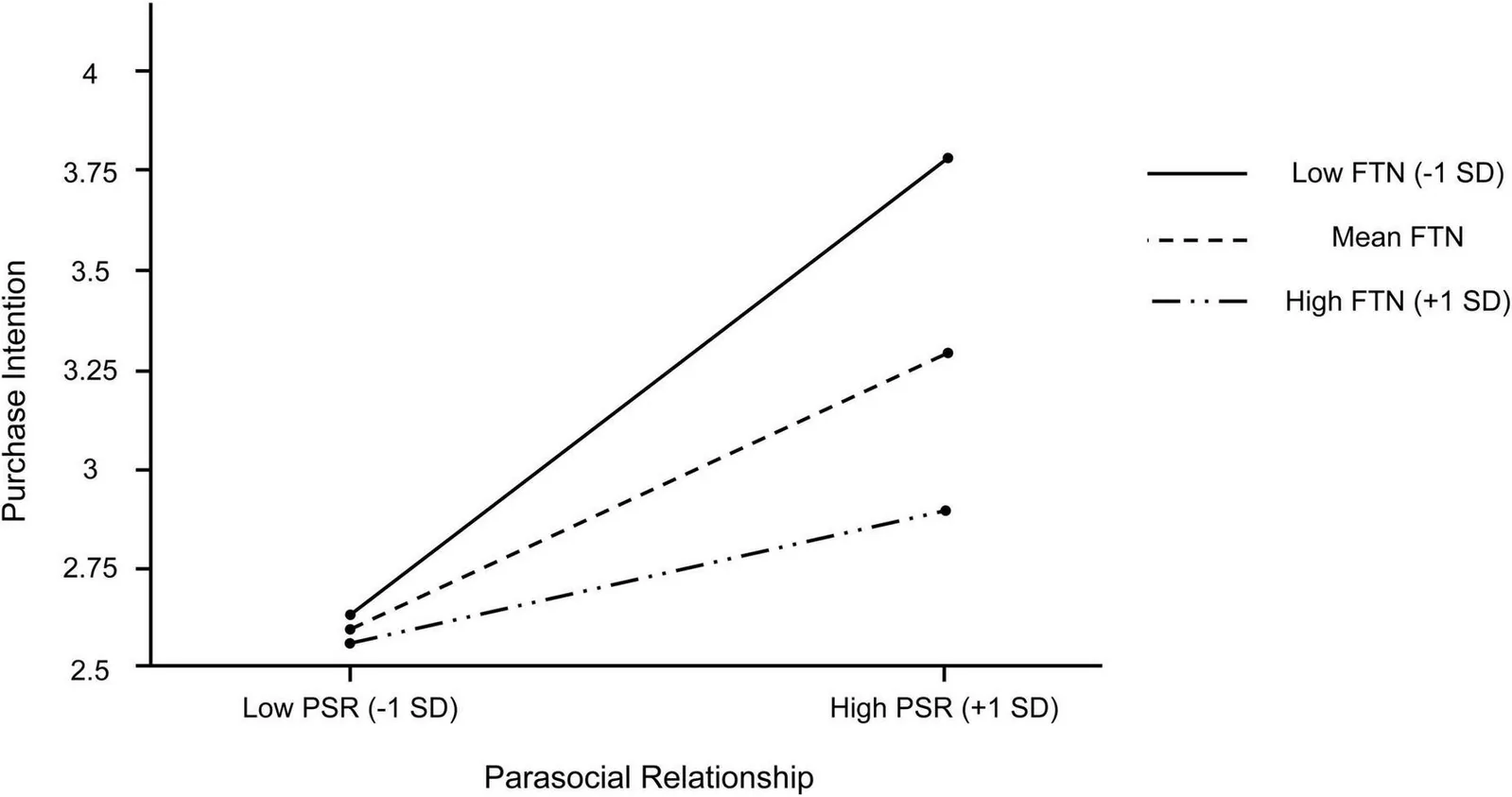
Moderating effect of food technology neophobia on the association between parasocial relationships and purchase intention.

A simple slope analysis was subsequently conducted to examine the association between parasocial relationships and purchase intention at different levels of food technology neophobia. As shown in Table 6, when food technology neophobia was low, parasocial relationships were strongly and positively associated with purchase intention (*B* = 0.563, 95% CI = [0.439, 0.688]). At the mean level of food technology neophobia, the association remained positive and significant (*B* = 0.373, 95% CI = [0.282, 0.464]). When food technology neophobia was high, the positive association between parasocial relationships and purchase intention was substantially weaker but remained statistically significant (*B* = 0.182, 95% CI = [0.045, 0.319]). As illustrated in Figure 2, the slope of the relationship between parasocial relationships and purchase intention was steepest at low levels of food technology neophobia and became progressively flatter as food technology neophobia increased. This pattern provides a more intuitive illustration of the negative moderating effect, showing that the positive influence of parasocial relationships on purchase intention was strongest among consumers with relatively low food technology neophobia and weakest among those with relatively high food technology neophobia. These results confirm the negative moderating role of food technology neophobia and therefore support H4.

**TABLE 6:** Simple slope analysis of the moderating effect of food technology neophobia.

Path	Level of FTN	*B*	SE	*t*	*p*	95% CI
PSR → PI	Mean	0.373	0.046	8.045	0.000	[0.282, 0.464]
	High (+1 SD)	0.182	0.070	2.617	0.009	[0.045, 0.319]
	Low (−1 SD)	0.563	0.063	8.878	0.000	[0.439, 0.688]

PSR, parasocial relationship; PI, purchase intention; FTN, food technology neophobia; SD, standard deviation; SE, standard error; CI, confidence interval.

## 5 Discussion

### 5.1 Main findings

This study examined how virtual influencers’ social attractiveness, perceived authenticity, and self-disclosure were associated with parasocial relationships and how these relationships were linked to consumers’ purchase intention toward upcycled foods recommended by virtual influencer. All three characteristics were positively associated with parasocial relationships, which were subsequently positively associated with purchase intention. Parasocial relationships partially mediated the associations between each virtual influencer characteristic and purchase intention. Food technology neophobia further weakened the positive association between parasocial relationships and purchase intention. Taken together, these findings suggest that consumers’ responses to virtual influencer recommendations reflect both a relationship-building process and an evaluation of the production and processing methods used for the recommended food.

The direct-effect results indicate that social attractiveness, perceived authenticity, and self-disclosure contribute to parasocial relationships through distinct but complementary relational functions. Social attractiveness may increase consumers’ willingness to approach and continue following a virtual influencer because a likable and approachable digital persona makes repeated exposure more appealing (13, 37). Perceived authenticity supports the recognition of a coherent identity. Although consumers understand that virtual influencers are digitally created rather than real human beings, they still evaluate whether the persona, expressed values, and content remain natural and consistent over time (61). Self-disclosure provides personal information about everyday experiences, preferences, opinions, and emotions, thereby increasing perceived familiarity and reducing psychological distance (15, 62). These findings support the view that parasocial relationships with virtual influencers are not based solely on visual design or technological novelty. They also depend on whether the digital persona remains socially approachable, internally consistent, and personally knowable through repeated exposure (19).

Among the three characteristics, perceived authenticity showed the strongest standardized association with parasocial relationships, followed by social attractiveness and self-disclosure. This pattern highlights the importance of identity coherence in relationships with nonhuman digital figures. Because virtual influencers are deliberately created and commercially managed, consumers may be particularly attentive to inconsistencies between their established personas, stated values, and promotional content (63, 64). A virtual influencer that communicates in a natural and coherent manner may therefore be more readily perceived as a stable relational figure rather than as a replaceable advertising instrument. Social attractiveness remains important because it encourages initial approach and continued attention, while self-disclosure gives consumers additional opportunities to learn about the persona. The comparatively smaller coefficient for self-disclosure does not imply that it is unimportant. Instead, personalized disclosure may support relationship development most effectively when the disclosed content is consistent with an already coherent and credible identity (65, 66). The three characteristics should therefore be understood as complementary relational resources rather than competing indicators of virtual influencer effectiveness.

The mediation results further show that parasocial relationships constitute an important, but not exclusive, mechanism linking virtual influencer characteristics with purchase intention toward upcycled foods (10, 67). When consumers perceive a virtual influencer as familiar and psychologically close, its recommendation may be interpreted as advice from a meaningful relational source rather than as an isolated commercial message. This relational cue may be particularly relevant for upcycled foods because consumers may find it difficult to directly assess the origin and condition of recovered ingredients, the processing procedures, the nutritional quality, and the naturalness of the final product before purchase (68, 69). The significant indirect effects indicate that social attractiveness, perceived authenticity, and self-disclosure are associated with purchase intention partly because they help establish a psychological relationship through which the recommendation is interpreted (70). The remaining direct effects, however, indicate that parasocial relationships do not fully account for these associations. Other mechanisms, including recommendation credibility, product trust, perceived value, perceived influencer–product fit, and attention to the product message, may also contribute to consumers’ purchase judgments.

The moderation results identify food technology neophobia as an important boundary condition of relationship-based persuasion. The positive association between parasocial relationships and purchase intention was strongest among consumers with relatively low food technology neophobia and became progressively weaker as food technology neophobia increased (17, 50). Consumers may feel close to and appreciate the communication style of a virtual influencer while still expressing concerns about the recovery, treatment, and reprocessing of residual food materials. Such concerns may relate to perceived naturalness, food safety, and uncertain long-term health consequences. Relational closeness and technology-related concern therefore appear to operate simultaneously rather than as mutually exclusive explanations of consumer response. Notably, the association between parasocial relationships and purchase intention remained positive and significant even at a high level of food technology neophobia. This pattern indicates that technology-related resistance constrains, but does not eliminate, the relevance of parasocial relationships. It also suggests that food technology neophobia may depend less on the objective complexity of the production process than on how consumers interpret the transformation of underutilized food materials into new edible products (60). A favorable relationship with a virtual influencer can support acceptance, but it cannot substitute for transparent information about ingredient sourcing, processing procedures, product safety, and nutritional quality.

### 5.2 Theoretical contributions

This study makes several theoretical contributions to research on virtual influencer marketing and consumer acceptance of upcycled foods. First, it provides a relational framework for integrating social attractiveness, perceived authenticity, and self-disclosure. Previous studies have often examined these characteristics separately as favorable attributes associated with consumer responses. The present study instead distinguishes their complementary functions in relationship formation. Social attractiveness encourages approach and continued attention, perceived authenticity supports the recognition of a coherent and stable digital identity, and self-disclosure increases personal knowledge and perceived familiarity. This distinction moves beyond the simple accumulation of virtual influencer attributes by explaining how different characteristics contribute to separate stages or dimensions of relational development. The findings show that consumers’ connections with virtual influencers are jointly supported by social appeal, identity coherence, and personalized information rather than by a single general evaluation of the communication source.

Second, the study extends parasocial relationship theory to the marketing of upcycled foods by identifying parasocial relationships as a mechanism linking virtual influencer characteristics with purchase intention. Parasocial relationship research has traditionally focused on audiences’ psychological connections with media figures, celebrities, and human content creators. The current findings indicate that consumers can also develop meaningful one-sided relationships with digitally created personas and use these relationships when evaluating recommendations for an unfamiliar food innovation. This application is theoretically relevant because an upcycled food product may generate questions about ingredient origins, reprocessing, safety, nutritional quality, and naturalness. The significant indirect effects suggest that consumers do not respond only to the observable characteristics of virtual influencers; they also interpret product recommendations through the psychological relationships accumulated through repeated exposure. At the same time, the partial rather than full mediation indicates that parasocial relationships represent one important mechanism among several possible processes, leaving room for recommendation credibility, product trust, perceived value, and influencer–product fit to explain additional variance.

Third, the study identifies food technology neophobia as a boundary condition of relationship-based persuasion. Research on virtual influencers generally emphasizes how favorable communicator characteristics and relational closeness enhance consumer responses, whereas research on innovative foods tends to focus on product knowledge, perceived risk, naturalness, and resistance to unfamiliar technologies. By incorporating these perspectives into one model, this study shows that relational closeness and technology-related resistance can operate simultaneously. A strong parasocial relationship is positively associated with purchase intention, but this association becomes weaker as food technology neophobia increases. The persistence of a significant positive relationship even at a high level of food technology neophobia further indicates that technology-related concern constrains rather than completely eliminates relational influence. This finding refines parasocial relationship theory by showing that the persuasive relevance of psychological closeness depends partly on consumers’ evaluations of the recommended product category. It also contributes to upcycled food research by demonstrating that market acceptance is shaped not only by product-related evaluations but also by the relationship between consumers and the digital actors communicating the product.

### 5.3 Practical implications

The findings suggest that virtual influencer campaigns for upcycled foods should be treated as long-term relationship-building activities rather than isolated endorsement events. Social attractiveness can attract initial attention, but sustained parasocial relationships also depend on whether the influencer maintains a coherent identity and provides personally meaningful content over time. Firms should therefore align the influencer’s visual identity, stated values, communication style, and product endorsements. Self-disclosure may strengthen familiarity when it is integrated naturally into content about daily routines, food preferences, environmental concerns, or sustainable lifestyles rather than presented as repetitive promotional messaging.

Upcycled food recommendations should also fit the virtual influencer’s established content narrative. Influencers associated with sustainable consumption, healthy eating, food innovation, or responsible lifestyles can present upcycled products as part of ordinary consumption practices rather than as abstract technological innovations. At the same time, firms should not rely solely on the influencer’s appeal. Communication should clearly distinguish edible, recoverable food materials from spoiled or unsafe waste and explain ingredient origins, recovery procedures, safety inspection, processing, and quality control in accessible language. Traceability information, nutritional details, production standards, and third-party certification may further reduce uncertainty, while vague claims such as “made from waste” should be avoided because they may reinforce perceptions of contamination or low quality.

The moderating role of food technology neophobia indicates that communication should be adapted to consumers’ relative level of technology-related concern. For consumers with lower food technology neophobia, marketing may emphasize innovation, resource efficiency, food waste reduction, and the lifestyle appeal of the virtual influencer. Consumers with moderate levels may respond better when relationship-oriented content is combined with understandable explanations of ingredient recovery and processing, supported by short videos, process illustrations, or traceability links. For consumers with higher food technology neophobia, firms should prioritize transparent sourcing, standardized processing, safety testing, quality certification, and credible information from food scientists, nutrition professionals, or independent certification bodies. Highly futuristic language and exaggerated technological claims should be avoided because they may strengthen perceptions that the product is excessively processed or unnatural.

Successful virtual influencer marketing for upcycled foods therefore requires coordination between relationship management and product assurance. Virtual influencers can attract attention, create familiarity, and make sustainability messages more personally relevant, but these relational advantages should be supported by transparent and verifiable information about ingredient origins, processing procedures, safety, and nutritional quality. Combining a coherent digital persona with communication strategies suited to different levels of food technology neophobia may strengthen acceptance across a broader range of upcycled food products.

## 6 Limitations and future research

This study has several limitations that should be considered when interpreting its findings. First, respondents evaluated the virtual influencer with whom they were most familiar or whom they followed most frequently. This approach increased ecological relevance because participants based their evaluations on actual following experience rather than on an unfamiliar digital character selected by the researchers. However, it also introduced heterogeneity in the focal influencers. The evaluated virtual influencers may have differed in visual design, anthropomorphism, content category, platform presence, commercial orientation, interaction style, and frequency of product endorsement. Future studies could classify virtual influencers according to their main characteristics and compare the proposed relationships across different influencer types. Controlled experiments using the same virtual influencer for all participants could also manipulate authenticity cues, self-disclosure, social attractiveness, endorsement disclosure, or influencer–product fit to establish more precise causal evidence.

Second, the study used a standardized scenario involving an upcycled apple chip product made from edible apple peels and pulp remaining from juice production and fruit-processing activities. Presenting the same product description to all respondents improved consistency in the evaluation context, but the findings may not generalize to other upcycled food categories. Consumer responses may differ for products made from spent grains, vegetable residues, dairy by-products, or seafood-processing materials because these ingredients vary in familiarity, perceived naturalness, disgust sensitivity, processing intensity, and expected safety. Future research should compare several upcycled ingredients and final product forms to determine whether the effectiveness of virtual influencer recommendations depends on the characteristics of the focal food product.

Third, the survey did not directly measure respondents’ prior knowledge of, familiarity with, or previous consumption of upcycled foods. All participants received the same definition of upcycled foods and the same apple chip scenario before answering the focal questions, which helped reduce differences in their immediate understanding of the product. Nevertheless, pre-existing knowledge and experience may still have influenced how they interpreted the scenario and evaluated the recommendation. Future studies should measure objective and subjective product knowledge, familiarity, prior consumption, and perceived understanding. These variables could be included as controls or examined as moderators to determine whether consumers with different levels of experience respond differently to virtual influencer communication. Comprehension checks could also be incorporated to verify that respondents correctly understand the meaning of upcycled foods and the source of the recovered ingredients.

Finally, this study relied on cross-sectional self-reported data collected at a single point in time. Although the statistical assessment suggested that common method bias was unlikely to pose a serious threat, self-reported responses may still be affected by social desirability, recall error, and subjective judgment. The cross-sectional design does not establish temporal ordering and therefore does not support strong causal conclusions regarding the relationships among virtual influencer characteristics, parasocial relationships, and purchase intention. In addition, the dependent variable reflected purchase intention in an imagined recommendation scenario rather than actual purchasing behavior. Future studies could employ time-lagged or longitudinal designs to examine how repeated exposure contributes to the development of parasocial relationships over time. Scenario-based, laboratory, and field experiments could manipulate virtual influencer characteristics and food-technology information to test causal effects. Behavioral measures such as viewing duration, click-through behavior, information searches, product choice, willingness to pay, additions to shopping carts, and actual purchases would provide stronger evidence of whether favorable relational responses translate into real consumption of upcycled foods.

## Data Availability

The original contributions presented in the study are included in the article/Supplementary material, further inquiries can be directed to the corresponding authors.
