# Supplementary material for: When parasocial relationships meet food technology neophobia: understanding consumer responses to virtual influencer marketing of upcycled foods
Source: Front Nutr. 2026 Sep 10;13:1933051. doi: 10.3389/fnut.2026.1933051 (PMC13600996; doi:10.3389/fnut.2026.1933051)
Supplement: Supplementary file 1 [file Table_1.docx]

Appendix. Measurement items

| Construct | Code | Item |
| --- | --- | --- |
| Social Attractiveness | SA1 | I find this virtual influencer to be a likable digital character. |
|  | SA2 | I find it enjoyable to follow this virtual influencer. |
|  | SA3 | I would enjoy having a friendly interaction with this virtual influencer. |
|  | SA4 | This virtual influencer seems approachable to me. |
| Perceived Authenticity | PA1 | The persona presented by this virtual influencer feels authentic and natural. |
|  | PA2 | This virtual influencer presents a consistent persona across different content. |
|  | PA3 | The views and values expressed by this virtual influencer are consistent with its established persona. |
|  | PA4 | The content posted by this virtual influencer feels like a natural expression of its persona rather than something deliberately contrived. |
| Self-Disclosure | SD1 | This virtual influencer frequently shares content about its daily life. |
|  | SD2 | This virtual influencer frequently shares its interests, preferences, or lifestyle habits. |
|  | SD3 | This virtual influencer is willing to express its thoughts and feelings in its content. |
|  | SD4 | This virtual influencer frequently explains the reasons behind its choices or decisions. |
| Parasocial Relationship | PR1 | After following this virtual influencer for some time, I feel that I know it fairly well. |
|  | PSR2 | I feel a sense of psychological closeness to this virtual influencer. |
|  | PSR3 | Following this virtual influencer sometimes feels like keeping in touch with someone familiar. |
|  | PSR4 | I would miss the content of this virtual influencer if it stopped posting for a long time. |
|  | PSR5 | I care about the experiences, views, and future updates of this virtual influencer. |
| Purchase Intention | PI1 | I would buy the upcycled food recommended by this virtual influencer. |
|  | PI2 | I intend to buy the upcycled food recommended by this virtual influencer. |
|  | PI3 | I am interested in buying the upcycled food recommended by this virtual influencer. |
|  | PI4 | I am likely to buy the upcycled food recommended by this virtual influencer in the future. |
| Food Technology Neophobia | FTN1 | I believe that the benefits of new food technologies are often exaggerated. |
|  | FTN2 | I am concerned that new food technologies may reduce the natural qualities of food. |
|  | FTN3 | I am reluctant to try foods produced using new food technologies. |
|  | FTN4 | I generally feel uncertain about new food technologies. |
|  | FTN5 | I believe that adopting new food technologies too quickly may involve risks. |
|  | FTN6 | I am concerned that foods produced using new food technologies may have adverse long-term effects on health. |
